# Pharmacology and Therapeutics

**Published:** 1891-01

**Authors:** 


### PHARMACOLOGY AND THERAPEUTICS.

### (227) FURTHER TRIALS OF OREXIN, CHLORALAMIDE, AND AMYLENE HYDRATE.

Dr. Umpfenbach records his experience with these drugs on patients in the lunatic asylum at Andernach (*Therap. Monatsh.*, October, 1890). Hydrochloride of orexin was administered in 3 to 4-grain doses in 25 cases; 4 of these had loss of appetite from various bodily illnesses, 6 were hysterical or hypochondriacal, 4 had loss of appetite from psychical depression, and 11 had complete loss of appetite with refusal of food. The first 10 of these got the orexin in pills, the other 15 in powder in soup or milk. The taste is bitter, but it was usually, although not always, taken without much complaint. Out of the first-mentioned 4, 3 were greatly improved; out of the 6, 5; and out of the second-mentioned 4, only 1. In the second class of 11, many had to be forcibly fed, but in 5 of these the improvement was so marked that they continued to eat of their own accord. In 6 there was no effect. No inconveniences were observed, although Penzoldt has observed burning in the œsophagus, and Podgorski singing in the ears, after orexin. The author concludes that orexin is a useful bitter tonic, although it does not succeed in every case. Chloralamide was given in 55 cases, dissolved in weak alcohol and syrup. He began with 30 grains at night, and rose to 90 grains per dose and per day. In paranoia, general paralysis, and excitement in epileptics, it usually acted well, while in general restlessness and in melancholia its hypnotic effects were *nil* or very slight. In a very few cases the sleep was disturbed by dreams, in others gastric irritation and purgation were observed, also excitement and skin eruptions. As a rule, however, no unpleasant effects ensued, and the drug could be taken for months without affecting the heart, the blood vessels, or the uro-genital system. The author concludes that chloralamide is a useful enough hypnotic; that its action, however, is not certain, and that it has no advantages over many other hypnotics such as chloral hydrate. Amylene hydrate has been recommended in epilepsy by Wildermuth (*Neurol. Centralbl.*, 15, 1889). The author tried it in 7 cases of epilepsy, in 5 to 8 gramme doses *per diem*. In two patients (who had about 9 to 11 epileptic attacks monthly), the fits remained absent as long as the drug was taken. In another case the effect was very transient. In the others there was absolutely no improvement. The drug had the great drawbacks of causing sleeplessness and then excitement after its hypnotic effects had worn off. —*Supplement to the British Medical Journal, November*, 1890.

